# A Comparative Analysis of Various Machine Learning Algorithms to Improve the Accuracy of HbA1c Estimation Using Wrist PPG Data

**DOI:** 10.3390/s23167231

**Published:** 2023-08-17

**Authors:** Shama Satter, Tae-Ho Kwon, Ki-Doo Kim

**Affiliations:** Department of Electronics Engineering, Kookmin University, Seoul 02707, Republic of Korea; shama@kookmin.ac.kr (S.S.); kmjkth@kookmin.ac.kr (T.-H.K.)

**Keywords:** diabetes, glycated hemoglobin, machine learning, photoplethysmography

## Abstract

Due to the inconvenience of drawing blood and the possibility of infection associated with invasive methods, research on non-invasive glycated hemoglobin (HbA1c) measurement methods is increasing. Utilizing wrist photoplethysmography (PPG) with machine learning to estimate HbA1c can be a promising method for non-invasive HbA1c monitoring in diabetic patients. This study aims to develop a HbA1c estimation system based on machine learning algorithms using PPG signals obtained from the wrist. We used a PPG based dataset of 22 subjects and algorithms such as extreme gradient boosting (XGBoost), light gradient boosting machine (LightGBM), Categorical Boost (CatBoost) and random forest (RF) to estimate the HbA1c values. Note that the AC-to-DC ratios for three wavelengths were newly adopted as features in addition to the previously acquired 15 features from the PPG signal and a comparative analysis was performed between the performances of several algorithms. We showed that feature-importance-based selection can improve performance while reducing computational complexity. We also showed that AC-to-DC ratio (AC/DC) features play a dominant role in improving HbA1c estimation performance and, furthermore, a good performance can be obtained without the need for external features such as BMI and SpO_2_. These findings may help shape the future of wrist-based HbA1c estimation (e.g., via a wristwatch or wristband), which could increase the scope of noninvasive and effective monitoring techniques for diabetic patients.

## 1. Introduction

Diabetes is a chronic metabolic disorder that affects millions of people worldwide [1]. It is characterized by elevated blood glucose levels resulting from either insufficient insulin production or the body’s resistance to insulin. One of the key indicators of long-term glycemic control in diabetic patients is the glycated hemoglobin (HbA1c) level, which represents the average blood glucose concentration over the past 3 months [2]. The accurate and timely measurement of HbA1c levels is essential for effective diabetes management, prevention of complications, and improvements in patients’ quality of life [3].

Traditional methods for HbA1c estimation require blood samples to be analyzed in a laboratory setting, which is invasive and inconvenient for patients [4]. This has led to a growing interest in noninvasive and continuous monitoring techniques that can be easily integrated into patients’ daily lives [5]. One promising approach is the use of wrist photoplethysmography (PPG) data, which measure the changes in blood volume in the microvascular bed of tissue via light-emitting diodes (LEDs) and a photodetector (PD). PPG data have previously been employed for various health-monitoring applications such as heart rate, SpO_2_, and blood pressure estimation [6,7]. In addition, recent studies on glucose or HbA1c estimation using PPG signals have been published [8,9,10,11,12,13]. One of the previous studies used the simple Beer–Lambert-law-based model to estimate the HbA1c level in vivo [9]. Another study focused on estimations based on the photon diffusion theorem by considering both transmission- and reflection-type PPG signals [10]. These are mathematical and theory-based models developed to noninvasively estimate HbA1c values from fingertip PPG signals. In [11], machine learning algorithms (random forest and XGBoost) are employed to predict the actual value of blood glucose level from the extracted features. Here, related features are extracted from the PPG signals. In [12], features are extracted based on feature importance from the acquired PPG signals, and machine learning algorithms are used to estimate the glycated hemoglobin value from the extracted features. It should be noted that the results in the aforementioned references [8,9,10,11,12] are based on fingertip PPG data, whereas the proposed study focuses on wrist PPG data with wearable applications in mind.

In this study, we present a comparative analysis of various machine learning algorithms to improve the accuracy of HbA1c estimation using wrist PPG data. We investigate the performance of XGBoost [14], random forest (RF) [15], CatBoost [16], and LightGBM [17] algorithms on a dataset comprising 22 subjects’ PPG data. A leave-one-out cross-validation scheme was employed to validate the models [18], while a grid search technique was used to optimize the hyperparameters [19]. The performance of the model was assessed based on its ability to predict HbA1c levels from the PPG data. Our results indicate that the machine learning algorithms achieved varying levels of success in HbA1c estimation. In addition to applying feature-importance-based selection to each machine learning algorithm, we further improved the performance by taking the features extracted from the PPG signal, such as AC-to-DC ratio (AC/DC) values at various wavelengths. In general, blood flow affects the alternating current (AC) component of the PPG signal, while tissue properties and motion artifacts affect the direct current (DC) component. The findings of this study suggest that wrist PPG data combined with advanced machine learning techniques have the potential to provide a noninvasive and convenient alternative to HbA1c estimation, paving the way for more accessible diabetes monitoring and management.

The contributions of this study can be summarized as follows.

(1) We provide a thorough comparison of different machine learning techniques for estimating HbA1c using wrist PPG data. Here, we evaluate the relative performance of some of the most well-known algorithms: XGBoost, RF, LightGBM, and CatBoost.

(2) In order to cope with the substantial dependence of existing evaluation features based on PPG waveform characteristics, new evaluation features extracted from PPG signals, such as AC/DC values at various wavelengths, were proposed and the performance improvements were demonstrated.

(3) Performance was improved through feature importance-based selection and performance was compared and analyzed according to combinations of RGB wavelengths.

(4) To analyze the performance of HbA1c estimation utilizing machine learning algorithms, we compared several regression algorithms, and the groundwork for implementing the results in wrist PPG-based hardware is provided for completely non-invasive HbA1c estimation.

## 2. Methodology

**Figure 1:**
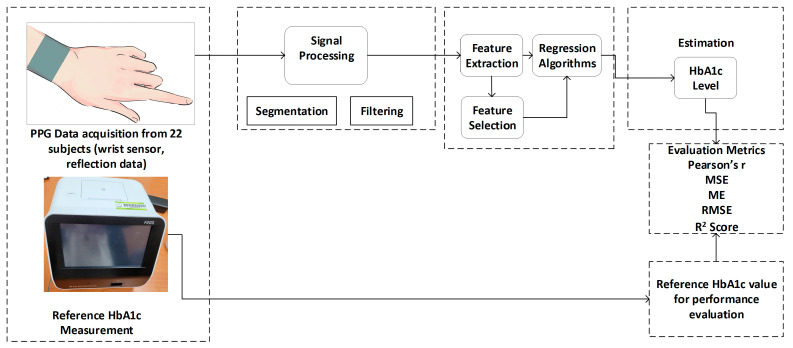
Proposed system architecture.

The wrist PPG signal data were acquired using a TMD3719-based [20] prototype with a photodetector (PD) and a low-power white LED of three wavelengths (465 nm, 525 nm, and 615 nm). Under the direct supervision of the institutional review board (IRB) of Kookmin University (IRB protocol number: KMU-202111-BR-286), Seoul, Republic of Korea, all of the data used to evaluate the experimental results were collected from 22 subjects. The 2 min raw data at the rate of 24 samples per second were then passed through the filter. The PPG signal was segmented into 3 s intervals, yielding approximately 72 samples for the 3 s interval. The segmented PPG signal was then fed through the system’s feature extraction module. We then conducted an experiment using the 15 features from the PPG signal as in [12], and also conducted an experiment using the dominant features selected based on feature importance. Due to the relatively low amount of data samples, all regression algorithms were optimized to ensure the selection of appropriate hyperparameters to prevent overfitting and the leave-one-out cross-validation (LOOCV) method was used for training each model. According to this method, one data point is left out from the dataset and the model is trained on the remaining data points. The left-out data point is then used as a validation set to test the model’s performance. This process is repeated for each data point in the dataset. Figure 1 shows a block diagram of the entire proposed system.

### 2.1. Hardware Device

**Figure 2:**
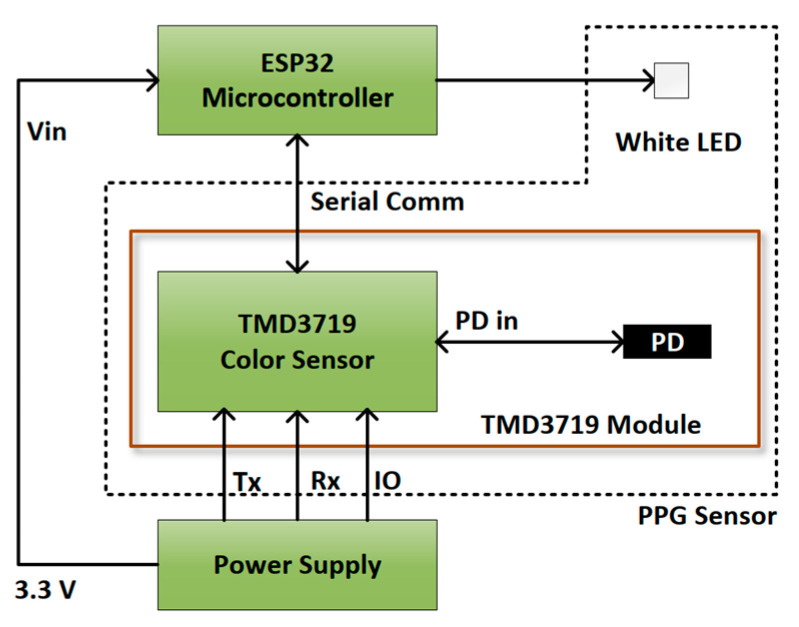
The block diagram of the proposed hardware system.

**Figure 3:**
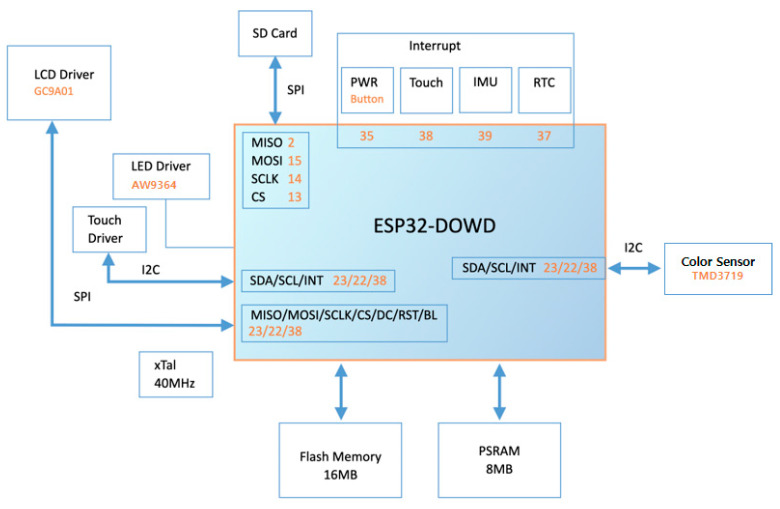
Detailed MCU peripheral circuit diagram.

**Figure 4:**
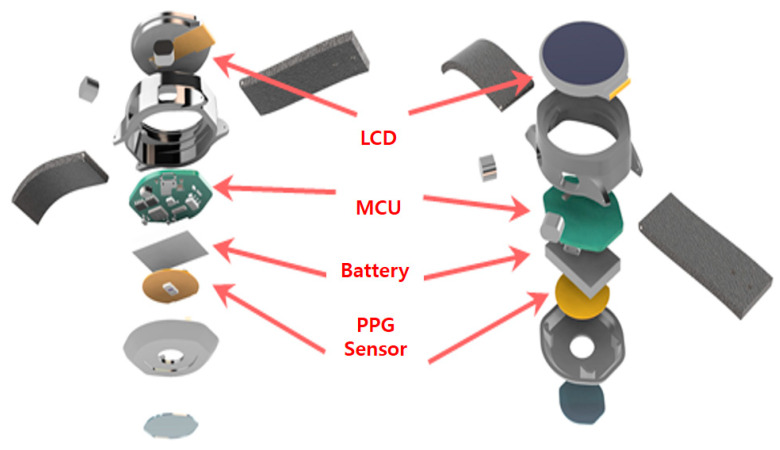
Structure diagram of the device.

The color sensor module TMD 3719 is used, in this study, for the purpose of color (RGB) sensing. The color sensor had three different filters on top of the sensor die: blue (465 nm), green (525 nm), and red (615 nm). An ESP32 microcontroller was used for communicating with the TMD 3719 module. We also used only one white LED (CLM3C-WKW) as a light source, instead of using three high-intensity light sources of different wavelengths. Figure 2 represents the block diagram of the proposed hardware system. Figure 3 presents a detailed microcontroller unit (MCU) peripheral circuit diagram for the MCU part in the block diagram of Figure 2. Finally, Figure 4 represents the structure diagram of the device.

### 2.2. Regression Models

Four regression models were fit and tested in this study. These four algorithms were chosen because of their strong performance in similar prediction tasks [21,22]. They are also known for their robustness and ability to capture complex patterns in data [23]. All regression algorithms were implemented with the Python programming language using the scikit-learn library. The possibility of overfitting was addressed through carefully considered hyperparameters and LOOCV was used for training each model. A description of the regression models used in this study is as follows.

#### 2.2.1. XGBoost

The XGBoost regressor is widely known as a powerful boosting algorithm. In this approach, decision trees are constructed in sequence, and the weights of all independent variables are computed before any of them are fed into the decision tree. In the second decision tree, additional weight is given to variables if they were mistakenly predicted by the first decision tree. In the end, the sum of these individual regressors will yield a reliable and accurate model [14]. Although XGBoost is capable of high performance, it frequently requires careful parameter-tuning to prevent overfitting and optimize the algorithm.

#### 2.2.2. Random Forest (RF)

To ensure that the model’s subsamples remain the same size as the original input, a random sampling technique called “replacement sampling” is used to extract from the dataset and fit a number of decision trees. The data are then averaged to prevent overfitting and improve prediction accuracy [15]. However, the model output is difficult to interpret and less effective in terms of speed and accuracy when compared to boosting algorithms.

#### 2.2.3. CatBoost

To compute the index of a leaf using bitwise operations, CatBoost generates “oblivious trees” with the constraint that all nodes at the same level must test the same predictor by applying the same conditions. The tree structure serves as a regularizer to find the optimal solution while avoiding overfitting, and the oblivious tree technique provides a simple fitting strategy and excellent CPU efficiency [16]. However, it may take a longer time to train.

#### 2.2.4. LightGBM

When generating decision trees, LightGBM uses an expansion strategy known as leaf-wise expansion. That is, if the condition is met, only one leaf is divided according to the gain. The training procedure for the standard gradient boosting decision tree can be sped up with the help of LightGBM [17]. Despite its tendency to overfit on small or noisy datasets, this risk can be mitigated through the cautious selection and tuning of hyperparameters. Given the size of our dataset, we were able to obtain a good balance between model complexity and overfitting risk using these parameters. The effectiveness of our model indicates that, even with a limited dataset, LightGBM can be a useful tool for predicting HbA1c from wrist PPG signals when designed properly.

### 2.3. Dataset Description

In this study, PPG-based data from 22 subjects were used along with the variables BMI, SpO_2_ and HbA1c. Statistics of the data set used are shown in Table 1. For the reference values, we measured the HbA1c and SpO_2_ values of the subjects using an invasive SD Biosensor F200 analyzer [24] and a MD300C26 fingertip pulse oximeter device [25], respectively.

### 2.4. PPG Signal Processing

**Figure 5:**
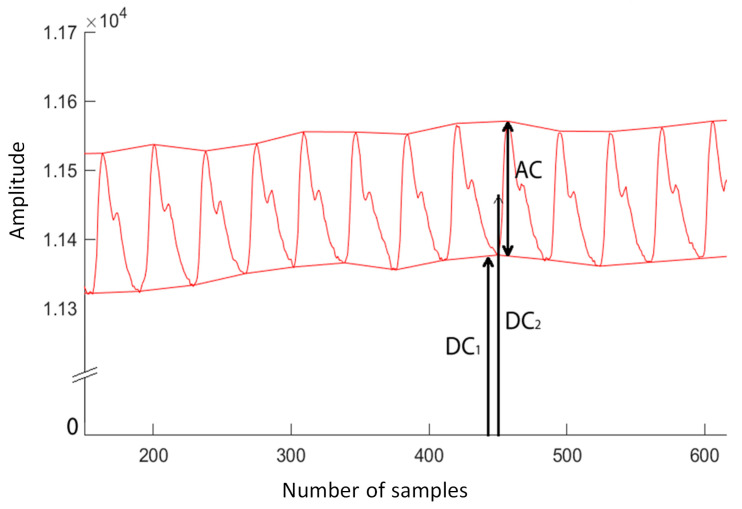
AC DC values of the typical PPG signal.

The raw PPG signal was filtered to remove high- and low-frequency noise and passed two-stage filtering. A second-order Butterworth low pass filter (LPF) with 8 Hz cutoff frequency was used to eliminate the high-frequency noise. The DC component is easily obtained by averaging the LPF output signal. After that, another 2nd-order high pass filter was used, with a cutoff frequency of 0.5 Hz, to remove the DC and respiratory components (less than 0.33 Hz). Baseline drift removal was also performed to keep the DC values constant. The AC and DC values in the PPG signal represent the pulsatile and the baseline (or static) component of the PPG signal, respectively. Figure 5 shows the AC and DC values of the typical PPG signal. The DC value can be defined as an average of valley values (DC_1_) or an average of intermediate values (DC_2_) of a peak value and a valley value.

### 2.5. Correct Peak and Valley Detection for Determining AC and DC Value from PPG Signal

To accurately obtain the AC and DC components of a PPG signal, it is important to accurately detect the peaks and valleys of the signal. We built an algorithm to accurately detect peaks and valleys. A pseudocode for determining AC and DC values from PPG signals is shown as Algorithm 1.
**Algorithm 1:** Pseudocode of determining AC and DC value from PPG signal.
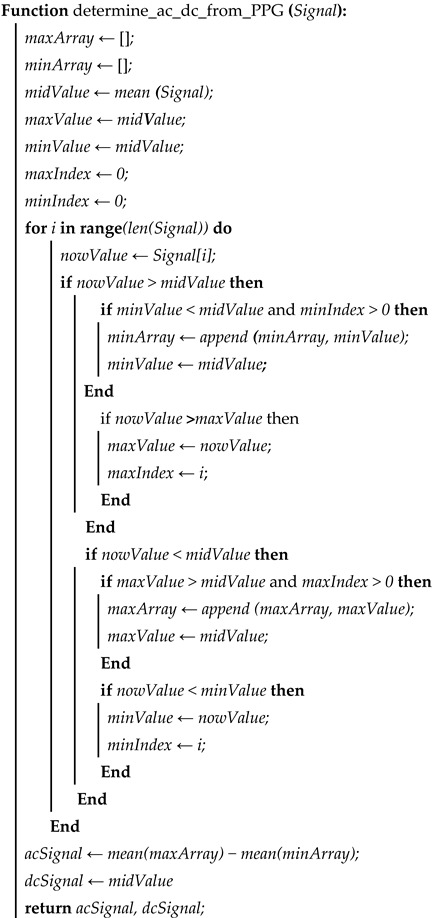


In the algorithm above, the function calculates the AC and DC components from a given input signal. The function then loops through each value in the input signal and updates the maximum and minimum values. Finally, the function computes the AC signal by taking the distance between the average of the maximum and the average of the minimum, and the DC signal by taking the average of the midpoint values, and returns these values.

### 2.6. Feature Extraction

The physiological attributes based on PPG signal and physical parameters can be regarded as features, and 15 significant and distinctive features were extracted from the PPG signal using time series feature extraction library (TSFEL) [11,12]. The 15 features are zero-crossing rate (ZCR), autocorrelation (ACR), kurtosis (kurt), variance (var), and mean of power spectral density (PSD); kurtosis (kurt), variance (var), mean, and skewness (skew) of Kaiser–Teager energy (KTE); kurtosis (kurt) and skewness (skew) of spectral analysis (spec); mean of wavelet analysis; autoregressive (AR) coefficients; skewness and sum of absolute difference (SAD). Among them, ZCR, ACR, and SAD are temporal features of the PPG signal, while the mean of wavelet analysis and PSD are spectral features. In addition, two other demographic features were considered: BMI and SpO_2_. The final feature vector can be obtained using Equation (1) for each frame of the PPG signal [12].

(1) $$X_{F}^{f}=\left[\begin{array}{c} s_{ZCR},s_{ACR},s_{PSD}^{kurt},s_{PSD}^{var},s_{PSD}^{mean},s_{KTE}^{kurt},s_{KTE}^{var},s_{KTE}^{mean},s_{KTE}^{skew},s_{spec}^{kurt},\;s_{spec}^{skew},\\ s_{wavelet}^{mean}\;,s_{AR}\;,s_{spo2},s_{skew},s_{SAD},\mathrm{BMI} \end{array}\right]$$

In particular, in this study, AC/DC values at different wavelengths calculated from PPG signals were considered as additional features to improve HbA1c estimation performance. The feature selection procedure is primarily based on feature importance. We used different techniques (Gini for random forest, gain-based method for XGBoost and CatBoost, both split- and gain-based method for LightGBM) to determine feature importance, and further improved the model by selecting the most significant features based on the importance metrics.

### 2.7. AC/DC Value as a Feature for Various Wavelengths

**Figure 6:**
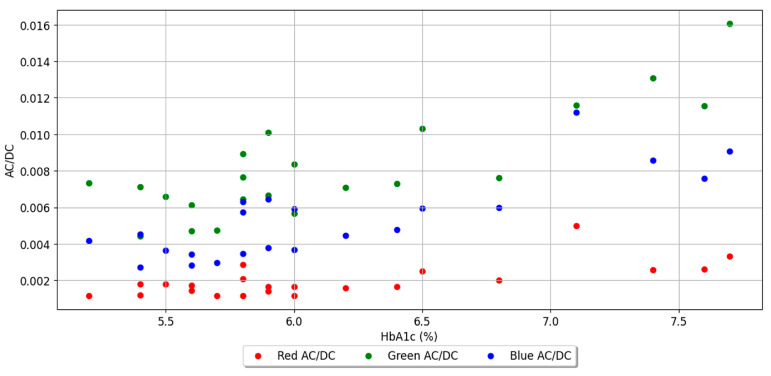
AC/DC values versus HbA1c for 22 subjects.

The AC/DC value represents the ratio of the pulsatile (i.e., AC) to the baseline or static (i.e., DC) component of the PPG signal. There could be an underlying relation between AC/DC value and HbA1c. However, there are few studies on this issue. One study demonstrated a proportional relationship between AC/DC value and glucose [26]. Another study showed a correlation between blood glucose levels and the AC/DC values of PPG signals [27]. Despite the lack of sufficient evidence to support a direct relationship between the AC/DC value and HbA1c levels, we obtained significantly better results using the AC/DC value as a feature. Figure 6 shows AC/DC values versus HbA1c for 22 subjects. In the case of the green wavelength, the AC/DC value is generally larger than those of the other two wavelengths (blue and red), and the results for all three wavelengths show a tendency to roughly increase according to the HbA1c value.

### 2.8. Importance-Based Feature Selection

**Figure 7:**
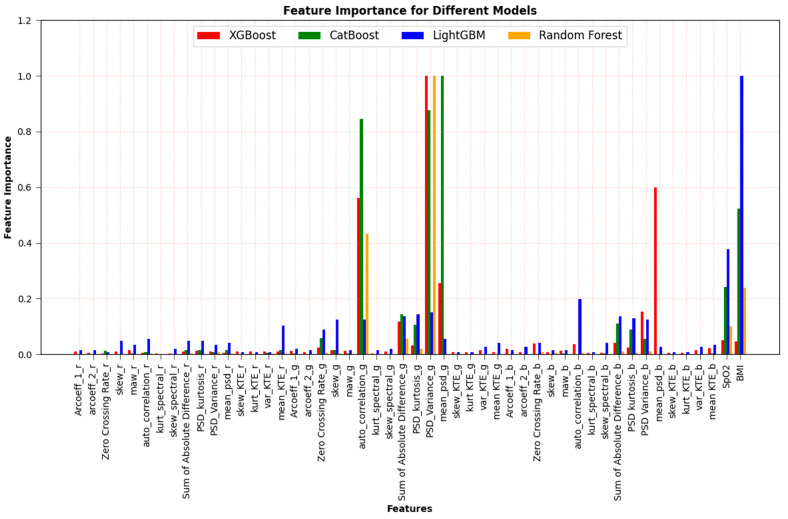
Feature selection based on feature importance for the 47 features (including external features).

**Figure 8:**
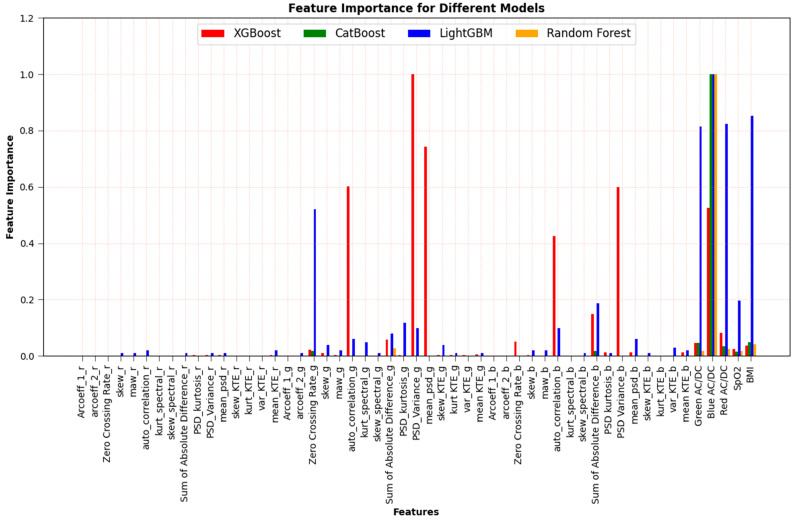
Feature selection based on feature importance for the 50 features (including AC/DC value as well as external features).

In reference [12], feature-importance-based selection was performed on 15 features obtained from the PPG signal. In this study, unlike the previous feature selection method, 15 features were considered for each wavelength, and feature selection was performed for each wavelength for a total of 47 features, including external features (BMI, SpO_2_). The corresponding feature importance plots for each of the four machine learning algorithms can be seen in Figure 7. Then, including AC/DC value at each wavelength as an additional feature, the feature importance plots for the total 50 features are also shown in Figure 8. To illustrate the efficacy of AC/DC values for the three wavelengths as new features, feature importance was compared with 47 existing features. As shown in the figure, AC/DC values are superior and can estimate the HbA1c more accurately.

The performance of all regression models was significantly improved using these new AC/DC features. Results are described in Section 3.2.

## 3. Results and Discussion

**Figure 9:**
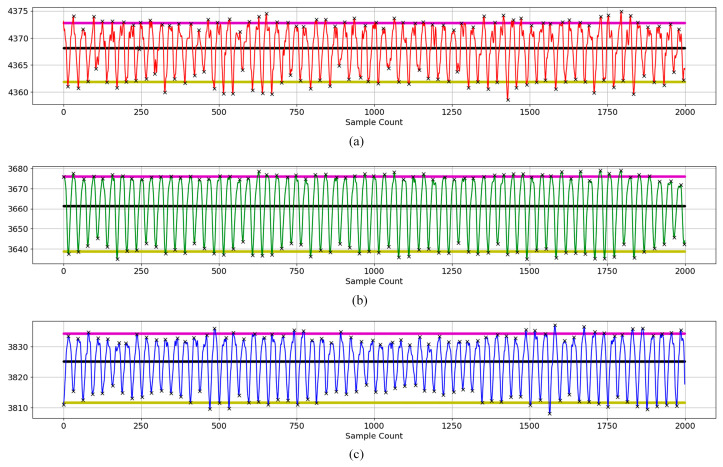
Peaks and valleys of a subject’s three-wavelength signal to determine AC and DC components (**a**) red wavelength, (**b**) green wavelength, (**c**) blue wavelength.

AC and DC values were calculated for a total of 22 subjects for three wavelengths: red, green, and blue. The AC/DC values were used as features in the machine learning algorithms. To efficiently and accurately detect the peaks and valleys of the PPG signal, the algorithm described in Algorithm 1 was used. An example of three wavelength signals after applying the algorithm is shown in Figure 9. The black line represents the average value of the signal, the magenta line represents the average value of the upper peaks, and the yellow line represents the average value of the lower valleys. The AC value is taken as the distance between the mean of the upper peaks and the mean of the lower valleys. DC value is taken as the average of intermediate and valley values. Black x marks are used to indicate peaks and valleys.

### 3.1. Performance When We Use 47 Features (without AC/DC Values)

In the dataset shown in Table 1, four machine learning algorithms were evaluated to estimate HbA1c. Algorithms included RF, XGBoost, LightGBM, and CatBoost. Pearson’s r values for the combination of wavelengths using the 47 features in Figure 7 are shown in Table 2. The reason for the poor Pearson’s r performance compared to the finger case in [12] is that the PD reception characteristic of the reflected signal from the wrist is inferior to that of the finger-reflected signal, and moreover, a finger width (FW) feature was additionally used in the case of a finger.

In Table 2, it can be observed that LightGBM performs relatively better in terms of HbA1c estimation compared to other algorithms. The key to its better performance might be the way it splits, as it uses a leaf-wise split approach instead of a level-wise split approach to make much more complex trees. Due to the overall unsatisfactory performance, the value of the AC-to-DC ratio is proposed as a new additional feature to improve the above results and is detailed in Section 3.2.

### 3.2. Performance When We Use 50 Features (Including AC/DC Features)

In addition to the 47 features in Figure 7, the AC/DC value of the PPG signal was added as a new feature. These features could be red wavelength AC/DC, green wavelength AC/DC and blue wavelength AC/DC values. The performance results are presented as Pearson’s r in Table 3, indicating a significant improvement in overall performance. We can also see that the performance improved more in the RF algorithm compared to other algorithms, because the AC/DC feature of the RF algorithm is relatively more dominant, as shown in Figure 8. The performance for the RGB combination obtained by the Pearson’s r value improved from 0.803 to 0.914, 0.796 to 0.904, 0.822 to 0.925 and 0.766 to 0.917 for the algorithms RF, XGBoost, LightGBM, and CatBoost, respectively, after AC/DC values were included as features.

### 3.3. Performance after Applying Feature-Importance-Based Selection

As shown in Figure 8, since the feature importance of AC/DC values is dominant, the four top features, in addition to AC/DC values, were selected for each wavelength of four machine learning algorithms based on the feature selection results in Figure 7 and Figure 8, and these are summarized in Table 4. Table 4 shows the dominant features of each wavelength (red, green, and blue), and the number in parenthesis indicates the order of importance.

Table 5 shows the Pearson’s r results after applying these selected important features. Note that, for the RGB combinations, the performance by Pearson’s r value after applying feature importance selection slightly improved, from 0.914 to 0.925, from 0.904 to 0.906, from 0.925 to 0.941, and from 0.917 to 0.921 for RF, XGBoost, LightGBM, and CatBoost algorithms, respectively. It did not show much improvement after feature selection; however, the performance is better, removing redundant features from the process. The training time also reduced after feature selection.

Table 6 shows the Pearson’s r for different algorithms using five selected features for different wavelength combinations without external features (BMI and SpO_2_). Overall, the performance of the algorithms used was relatively lower than when external features were applied; however, it was not much worse because AC/DC features dominate over external features, as shown in the feature importance plots of Figure 8. The results in Table 2, Table 3, Table 4, Table 5 and Table 6 provide valuable insight into the impact of PPG-based features on the model’s predictive capabilities.

For performance analysis, mean-squared error (MSE), mean error (ME), root-mean-squared error (RMSE), and $R^{2}\;$ score were used, in addition to Pearson’s r. The evaluation metric results for the various regression models are shown in Table 7.

As can be seen in Table 7, the LightGBM algorithm achieved the lowest MSE of 0.061 and RMSE of 0.246. It also achieved the highest $R^{2}\;$ score of 0.881. The results of various error metrics, including Pearson’s r, indicate that the LightGBM algorithm outperforms the other algorithms in terms of accuracy.

**Figure 10:**
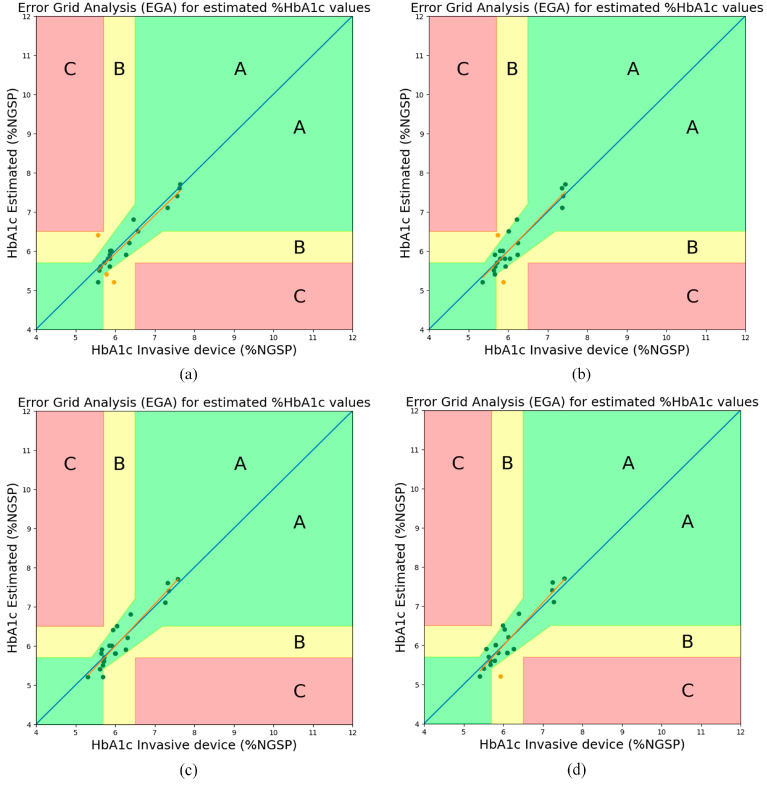
EGA plots for RGB with 7 selected features (5 features per wavelength, BMI, SpO_2_): (**a**) RF (**b**) XGBoost (**c**) LightGBM (**d**) CatBoost.

**Figure 11:**
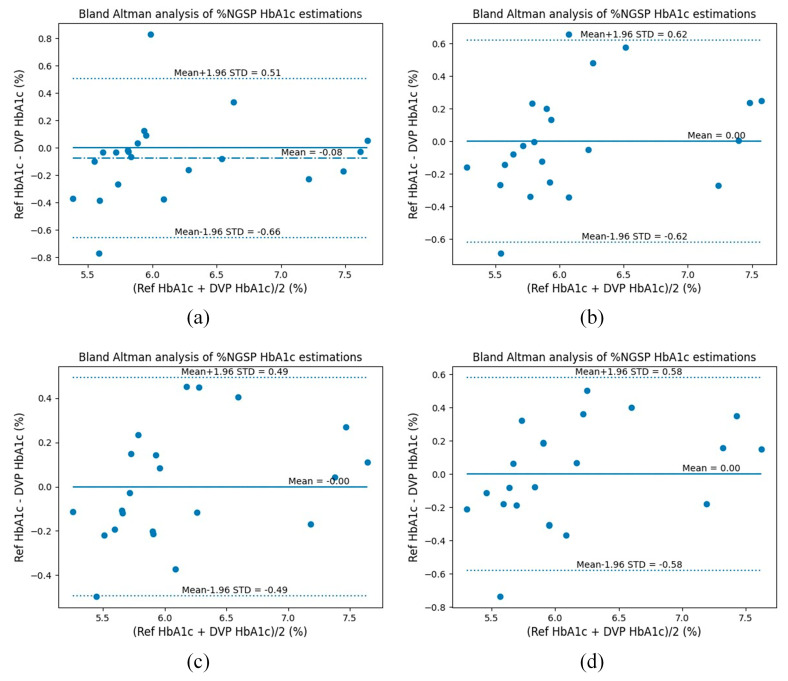
Bland–Altman analysis plots for RGB with 7 selected features (5 features per wavelength, BMI, SpO_2_): (**a**) RF (**b**) XGBoost (**c**) LightGBM (**d**) CatBoost.

To verify the clinical safety of our proposed noninvasive HbA1c estimation method, the most-used Clarke’s error grid analysis (EGA) [28] and Bland–Altman (B&A) analysis were conducted. Figure 10 and Figure 11 show the EGA and B&A plots, respectively, when using the selected features in Table 4. From the EGA plot, the data belonging to Zone A (includes clinically accurate data), Zone B (includes data outside of 20% of the reference, but would not lead to inappropriate treatment), and Zone C (includes data that would lead to inappropriate treatment) for each algorithm are summarized in Table 8.

Table 8 shows the area accuracy of the EGA plot when the selected features are applied. Consistent with the Pearson’s r performance in Table 5 and the evaluation metrics results in Table 7, the LightGBM algorithm shows the best performance; in this case, the area accuracy of the EGA plot was 100% and 0% for zone A and zone B, respectively.

Also, from the Bland–Altman analysis, the bias in the results and the corresponding limit of agreement for each algorithm are shown in Table 9. From Table 9, the bias of all four algorithms is comparatively small, with RF having the largest bias at −0.075 ± 0.296 and LightGBM having the smallest at 0.001 ± 0.252. Bias refers to the average difference between the algorithm’s predicted values and the actual values. A bias near to zero indicates that, on average, the algorithm’s predictions are close to the actual values. The Bland–Altman method also calculates the 95% limits of agreement as the mean difference (precisely, 1.96 standard deviation (STD)). The greater the agreement, the smaller the range between these two limits. All four algorithms have comparatively small limits of agreement, with LightGBM’s limit of agreement being the lowest, at 0.49.

A comparison of the error analysis findings with AC/DC as a feature indicated that significant improvements in performance are possible when AC/DC is employed. The AC-to-DC ratio of the PPG signal is a significant feature because it provides insight into the pulsatile and non-pulsatile components of the blood volume changes in the microvascular layer of the tissue being measured.

## 4. Conclusions

In this study, we presented an efficient and non-invasive HbA1c measurement system based on machine learning algorithms that utilizes the wristband PPG signal as well as physical parameters such as BMI and SpO_2_. We employed several regression models (RF, XGBoost, LightGBM, CatBoost) to estimate HbA1c levels for a PPG-based dataset of 22 subjects, and our results demonstrated that the inclusion of PPG-based features such as the AC/DC values of three wavelengths significantly improved the accuracy of the model. The performance for the RGB combination by the Pearson’s r value improved from 0.803 to 0.914, 0.796 to 0.904, 0.822 to 0.925 and 0.766 to 0.917 for the algorithms RF, XGBoost, LightGBM, and CatBoost, respectively, after AC/DC values were included as features. We also showed that feature importance-based selection can improve performance while reducing computational complexity. As shown in Table 5, the overall performance was slightly improved by Pearson’s r value after applying feature-importance selection by removing the redundant features in the process. The results of various error metrics, including Pearson’s r, indicate that the LightGBM algorithm outperforms the other algorithms in terms of both accuracy and predictive power. The LightGBM algorithm achieved the lowest MSE of 0.061 and RMSE of 0.246, and also achieved the highest $R^{2}\;$ score of 0.881. Finally, EGA and B&A analysis were performed to verify the clinical safety of the proposed non-invasive HbA1c estimation method. Consistent with the Pearson’s r performance in Table 5 and the evaluation metrics results in Table 7, the LightGBM algorithm showed the best performance; in this case, the area accuracy of the EGA plot was 100% and 0% for zone A and zone B, respectively. By calculating the 95% limits of agreement through B & A analysis, we showed that all four algorithms have comparatively small limits of agreement, with LightGBM’s limit of agreement being the lowest at 0.49.

Our findings in this study suggest that the proposed noninvasive HbA1c measurement system has the potential to provide accurate and reliable measurements of HbA1c levels, which may have significant clinical implications for diabetic patients. Although our study provides a promising proof-of-concept, further validation with a larger sample size is needed to fully evaluate the performance of the proposed system.

## Figures and Tables

**Table 1:** Statistics of the dataset used.

Measurement	BMI	SpO_2_ (%)	HbA1c
Min Max	19.50 30.52	96 99	5.2 7.7
Mean ± SD	25.53 ± 2.77	97.75 ± 0.79	6.10 ± 0.73

**Table 2:** Pearson’s r of different algorithms by using 47 features for all possible wavelength combination of three wavelengths, including BMI and SpO_2_.

Combination of Wavelengths	RF	XGBoost	LightGBM	CatBoost
R	0.273	0.559	0.593	0.514
G	0.735	0.786	0.808	0.796
B	0.752	0.767	0.770	0.710
RG	0.744	0.784	0.815	0.760
RB	0.731	0.711	0.761	0.713
GB	0.794	0.793	**0.826**	**0.780**
RGB	**0.803**	**0.796**	0.822	0.766

Best values are in boldface font.

**Table 3:** Pearson’s r of different algorithms by using 50 features for all possible wavelength combinations.

Combination of Wavelengths	RF	XGBoost	LightGBM	CatBoost
R	0.748	0.789	0.856	0.827
G	0.744	0.871	0.886	0.857
B	0.895	0.884	0.899	0.878
RG	0.747	0.867	0.833	0.858
RB	0.896	0.899	0.910	0.908
GB	0.876	0.879	0.914	0.905
RGB	**0.914**	**0.904**	**0.925**	**0.917**

Best values are in boldface font.

**Table 4:** Selected features based on feature importance for each ML algorithm.

Algorithm	Red	Green	Blue	Demographic Features
**RF**	Red AC/DC (4), PSD variance (12), autocorrelation (18), sum of absolute difference (19), mean KTE (20)	Sum of absolute difference (3), green AC/DC (6), PSD variance (7), autocorrelation (8), PSD Kurtosis (9)	Blue AC/DC (1), PSD variance (10), sum of absolute difference (11), zero-crossing rate (13), autocorrelation (14)	BMI (2), SpO_2_ (5)
**XGBoost**	Red AC/DC (8), mean PSD (23), mean KTE (24), PSD kurtosis (25), PSD variance (26)	PSD variance (1), Mean PSD (2), autocorrelation (3), sum of absolute difference (9), green AC/DC (11)	PSD variance (4), blue AC/DC (5), autocorrelation (6), sum of absolute difference (7), zero-crossing rate (10)	BMI (12), SpO_2_ (13)
**LightGBM**	Red AC/DC (3), mean KTE (22), autocorrelation (23), mean PSD (24), mean absolute wavelet (28)	Green AC/DC (4), zero-crossing rate (5), PSD kurtosis (8), PSD variance (10), sum of absolute difference (11)	Blue AC/DC (1), sum of absolute difference (7), autocorrelation (9) mean PSD (12), KTE variance (17)	BMI (2), SpO_2_ (6)
**CatBoost**	Red AC/DC (4), sum of absolute difference (15), PSD Kurtosis (17), mean KTE (18), mean PSD (23)	green AC/DC (3), mean PSD (6), PSD variance (7), autocorrelation (8), sum of absolute difference (10)	Blue AC/DC (1), sum of absolute difference (9), PSD kurtosis (12), autocorrelation (14), mean PSD (16)	BMI (2), SpO_2_(5)

**Table 5:** Pearson’s r for different algorithms using selected 7 features (including BMI and SpO_2_) for different wavelength combination.

Combination of Wavelengths	RF	XGBoost	LightGBM	CatBoost
R	0.736	0.766	0.859	0.831
G	0.725	0.891	0.887	0.832
B	0.918	0.881	0.906	0.857
RG	0.829	0.901	0.927	0.890
RB	0.924	0.901	0.890	0.888
GB	0.914	0.896	0.920	0.910
RGB	**0.925**	**0.906**	**0.941**	**0.921**

Best values are in boldface font.

**Table 6:** Pearson’s r for different algorithms using five selected features (excluding BMI and SpO_2_) for different wavelength combinations.

Combination of Wavelengths	RF	XGBoost	LightGBM	CatBoost
R	0.644	0.758	0.788	0.678
G	0.744	0.853	0.644	0.825
B	0.873	0.826	0.864	0.814
RG	0.786	0.861	0.858	0.769
RB	0.876	0.854	0.889	0.801
GB	0.909	0.870	0.896	0.882
RGB	**0.914**	**0.890**	**0.929**	**0.890**

Best values are in boldface font.

**Table 7:** Evaluation metrics for different algorithms using selected features for RGB wavelength combinations.

Metrics	RF		XGBoost		LightGBM		CatBoost	
	w/External Features	w/o External Features	w/External Features	w/o External Features	w/External Features	w/o External Features	w/External Features	w/o External Features
MSE	0.074	0.085	0.093	0.109	**0.061**	0.074	0.076	0.141
ME	−0.012	−0.073	0.024	0.035	**−0.004**	0.009	0.014	0.048
RMSE	0.271	0.292	0.305	0.329	**0.246**	0.272	0.277	0.375
$R^{2}\;$ Score	0.856	0.834	0.818	0.787	**0.881**	0.861	0.850	0.725

Best values are in boldface font.

**Table 8:** Zone accuracy of EGA plots for different algorithms using selected features for RGB wavelength.

Type	Zone		
	A	B	C
RF	86% (19)	14% (3)	0% (0)
XGBoost	91% (20)	9% (2)	0% (0)
LightGBM	**100% (22)**	**0% (0)**	0% (0)
CatBoost	95% (21)	5% (1)	0% (0)

Best values are in bold font. Number in parenthesis indicates the number of subjects belonging to the zone.

**Table 9:** The bias of the results and the corresponding limit of agreement for each algorithm using 7 selected features (5 features per wavelength, BMI and SpO_2_) for RGB wavelength.

Algorithm	Bias	95% Limit of Agreement (1.96 STD)
RF	−0.075 ± 0.296	−0.66 to 0.51
XGBoost	0.001 ± 0.278	−0.62 to 0.62
LightGBM	**0.001 ± 0.252**	**−0.49 to 0.49**
CatBoost	0.001 ±0.297	−0.58 to 0.58

Best values are in boldface font.

## Data Availability

The dataset used in this research is available upon a valid request to any of the authors of this research paper.
